# Short-term Diesel Exhaust Exposure Results in Neuroinflammation and White Matter Injury

**DOI:** 10.1093/geroni/igab046.2421

**Published:** 2021-12-17

**Authors:** Krista Lamorie-Foote, Kristina Shkirkova, Qinghai Liu, Constantinos Sioutas, Todd Morgan, Caleb Finch, William Mack

**Affiliations:** 1 University of Southern California - Keck School of, Los Angeles, California, United States; 2 University of Southern California, Los Angeles, California, United States; 3 Keck School of Medicine of the University of Southern California, Los Angeles, California, United States

## Abstract

Ambient air pollution (AAP) exposure is associated with white matter injury and cognitive decline in older adults(Chen et al. 2020,Erickson et al. 2020). Neuroinflammation and oxidative stress may contribute to this white matter injury. Diesel exhaust particulate matter (DEP) is a neurotoxic component of AAP.This study characterizes the time course by which neuroinflammation/oxidative stress occurs and results in white matter injury following DE exposure in a murine model. DEP (Sigma) was re-aerosolized for exposure. Mice were exposed to 100 µg/m3 DEP or filtered air (FA) for 5 hours (n=8/group), 100 hours (n=6/group), or 200 hours (n=6/group). Immunohistochemical analysis of degraded myelin basic protein (dMBP), a marker of myelin damage, was performed. Neuroinflammation and oxidative stress were assessed by histological analysis of complement C5a, an anaphylatoxin, and 4-Hydroxynonenal (4-HNE), a marker of lipid peroxidation.dMBP integrated density was increased in the corpus callosum of DEP mice at 5 (p<0.01), 100 (p<0.01), and 200 hours (p<0.001) compared to FA mice.C5a integrated density was increased in the corpus callosum of DEP mice at 5 (p<0.01), 100 (p<0.01), and 200 hours (p<0.01) compared to FA mice. 4-HNE integrated density was increased in the corpus callosum of DEP mice at 5 (p<0.001), 100 (p=0.001), and 200 hours (p<0.001) compared to FA mice. Neuroinflammation and oxidative stress are upregulated with associated white matter injury in the corpus callosum after 5 hours of DEP exposure.Short-term DEP exposure activates inflammatory/oxidative stress pathways, which may contribute to the pathogenesis of white matter injury.Erickson et al. 2020,PMID:32182984; Chen et al. 2020,PMID:32669395.

